# Supplementary material for: Calcium is an essential cofactor for metal efflux by the ferroportin transporter family
Source: Nat Commun. 2018 Aug 6;9:3075. doi: 10.1038/s41467-018-05446-4 (PMC6079014; doi:10.1038/s41467-018-05446-4)
Supplement: Supplementary file 1 — Supplementary Information [file 41467_2018_5446_MOESM1_ESM.pdf]

Supplementary Information for

# Calcium is an essential cofactor for metal efflux by the ferroportin transporter family

Deshpande et al.

**This PDF file includes:**

Supplementary Figures 1-11

Supplementary Table 1

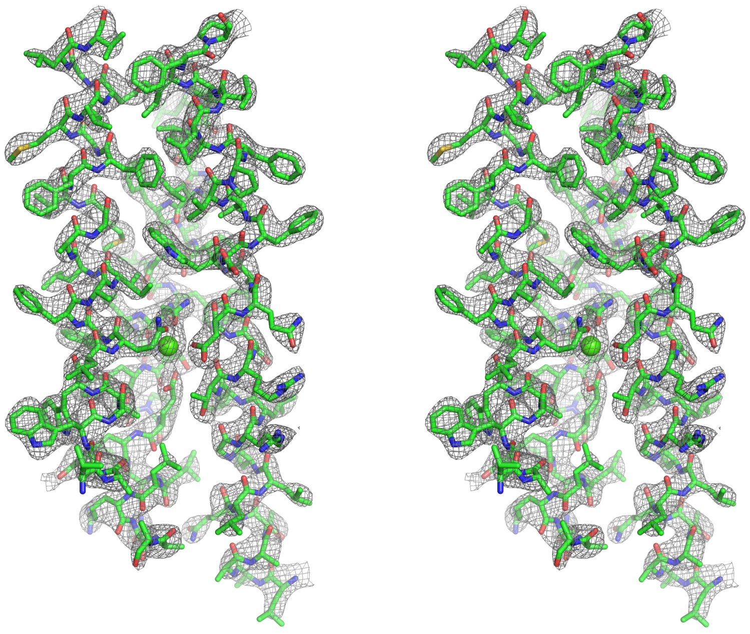

**Supplementary Figure 1. Stereo image of representative electron density for BbFpn.** Stereo image of the N-terminal domain of BbFpn (green), with $2mF_o$-$DF_c$ electron density (grey) contoured at 1.5σ.

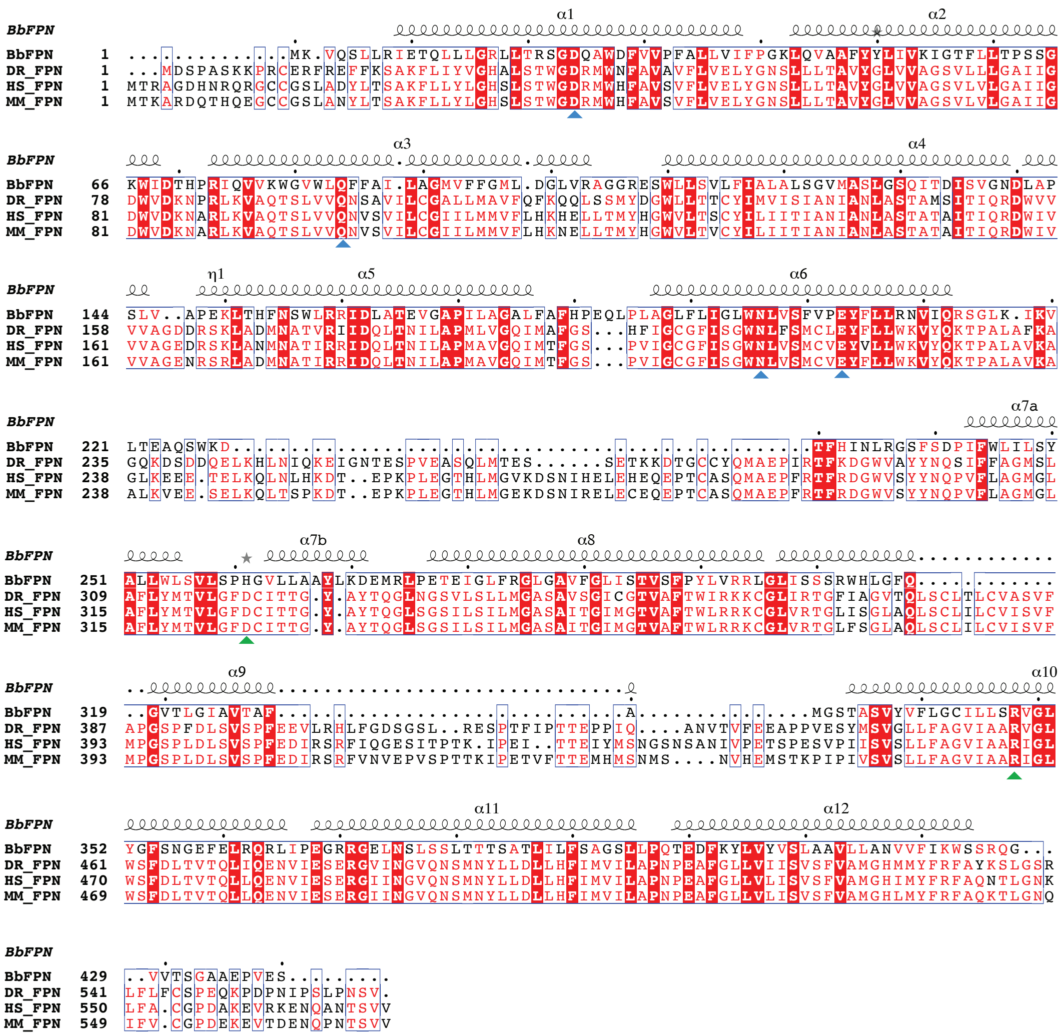

**Supplementary Figure 2. Sequence conservation between Fpn proteins.** Amino acid sequences for bacterial Fpn (BbFPN; *Bdellovibrio bacteriovorous*), zebrafish Fpn (DR_FPN; *Danio rerio*), human Fpn (HS_FPN), and mouse Fpn (MM_FPN). Secondary structure elements for α-helices are shown based on the structure of BbFpn (PDB entry 5AYN). Fully conserved residues are shown with red panels, and red letters for partially conserved. Residues implicated in $Ca^{2+}$-ion coordination are indicated with blue triangles, whilst putative substrate ligands are indicated by green triangles.

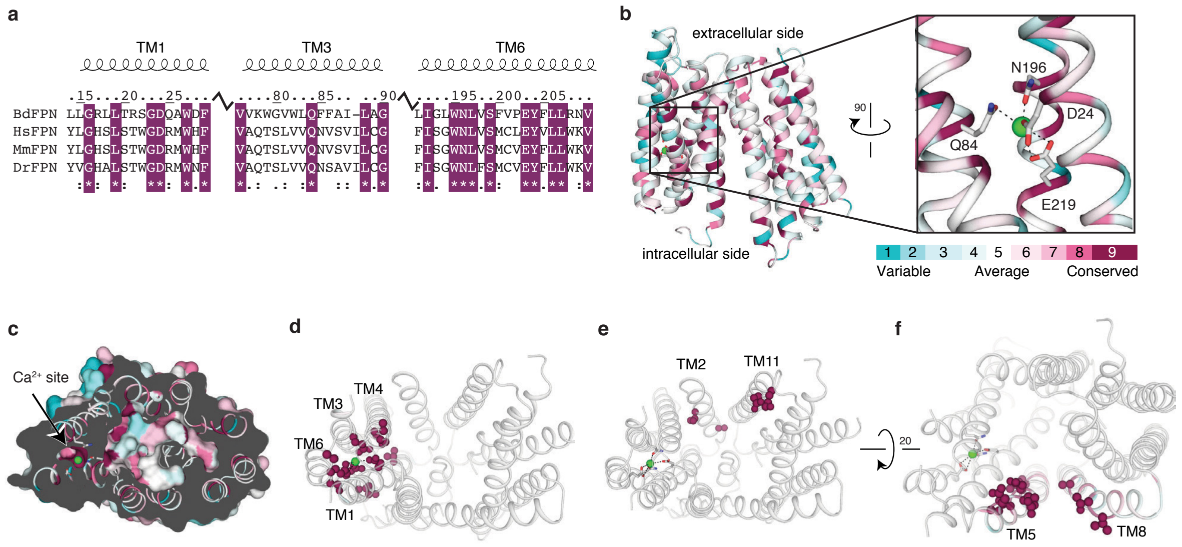


**Supplementary Figure 3. Sequence conservation in and around the $Ca^{2+}$ site in Fpn proteins. a** Sequence alignment of the $Ca^{2+}$ site, species names as in Supplementary Data Figure 2. **b** Overall structure with the $Ca^{2+}$ site enlarged (right). Figure is coloured from teal (most variable) to magenta (most conserved) on the basis of ConSurf analysis (http://consurfdb.tau.ac.il/). $Ca^{2+}$ ion is shown as a green sphere, and ligands are illustrated in ball-and-stick. **c** Surface rendering and colouring according to ConSurf with a view from the intracellular side of the membrane. The $Ca^{2+}$ ion is located in a conserved occluded pocket. **d** View as in **c**, with the conserved residues in the 4 helixes (TMs 1, 3, 4, and 6) surrounding the $Ca^{2+}$ site illustrated as purple spheres. The conserved residues in these helices are predominantly situated in and around the $Ca^{2+}$-site. **e-f** View from the intracellular side. Other conserved residues in the N-terminal domain are predominantly located on TM2 and TM5, which forms the main 'hinge' region between the two 6TM domains.

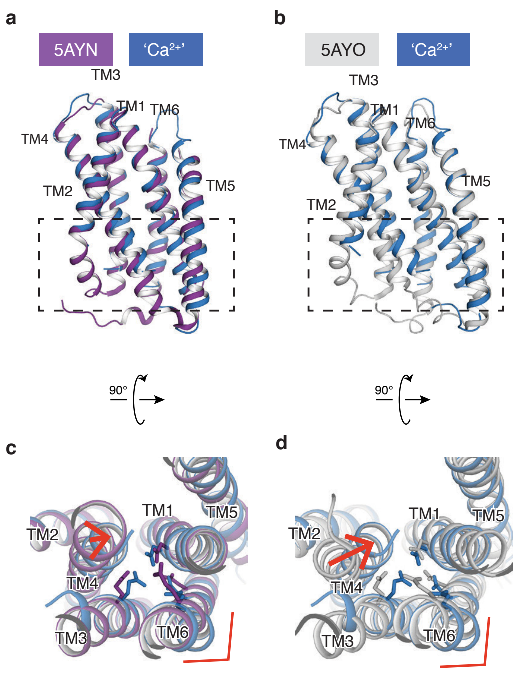


**Supplementary Figure 4. Conformational changes in the N-terminal domain. a** Superposition of $Ca^{2+}$ bound N-terminal domain with previously determined outward facing conformation (PDB entry 5AYN; purple) and **b** inward facing conformation (PDB entry 5AYO; grey). **c-d** View from the intracellular side, with the structural changes highlighted with red arrows and lines.

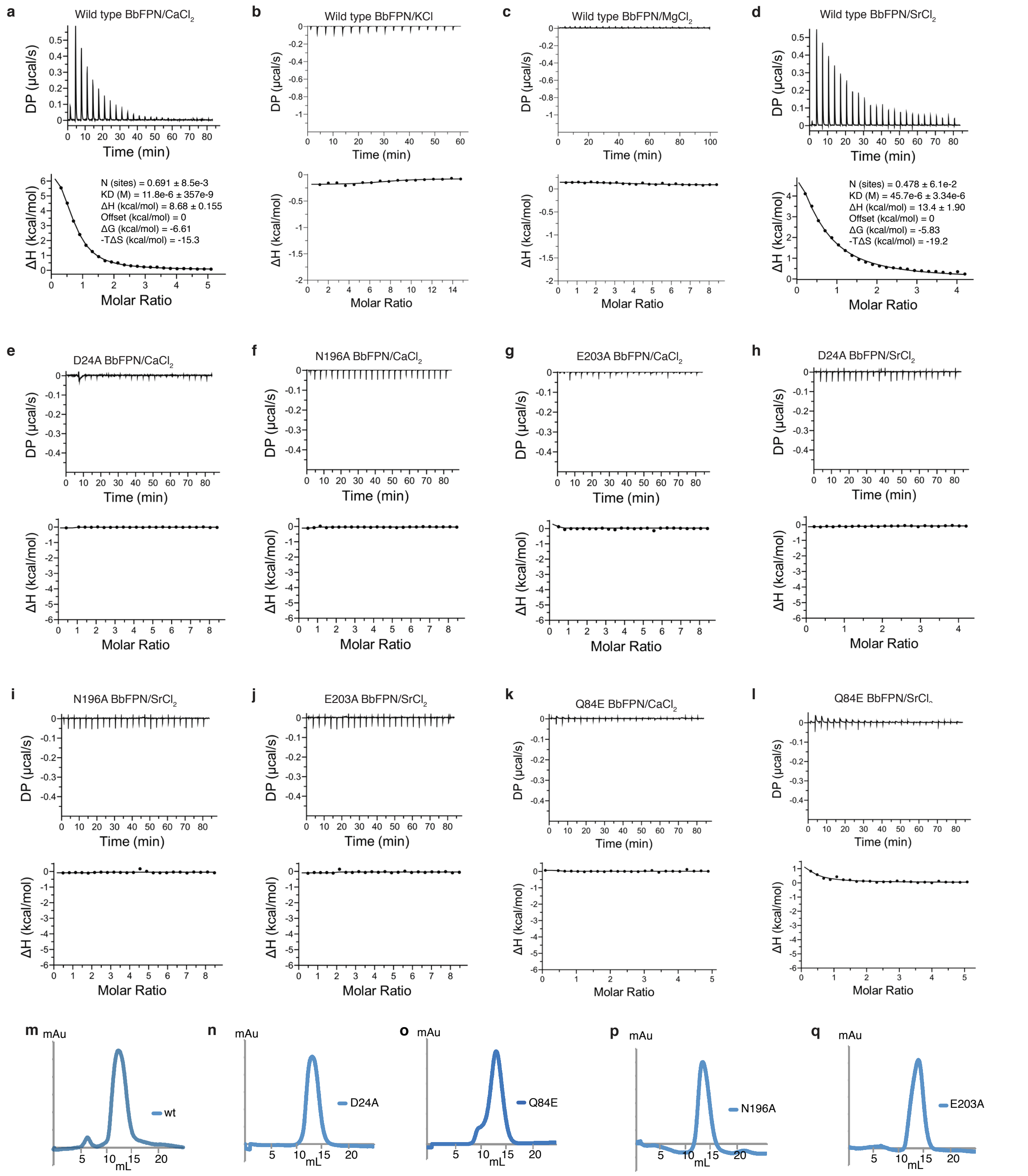


**Supplementary Figure 5. Isothermal titration calorimetry data. a-d** ITC isotherms of $Ca^{2+}$, $K^{+}$, $Mg^{2+}$, and $Sr^{2+}$ to wild-type BbFpn, respectively. **e-g** ITC measurements of $Ca^{2+}$ binding to D24A, N196A, E203A mutants of BbFpn. **h-j** ITC measurements of $Sr^{2+}$ binding to D24A, N196A, E203A mutants of BbFpn. **k-l** ITC measurements of $Ca^{2+}$ and $Sr^{2+}$ binding to Q84E. **m-q** Size exclusion chromatograms of wild type and D24A, Q84E, N196A, E203A mutants of BbFpn. Samples were run on a Superdex 200 10/300GL (GE Lifesciences), and A280 monitored.

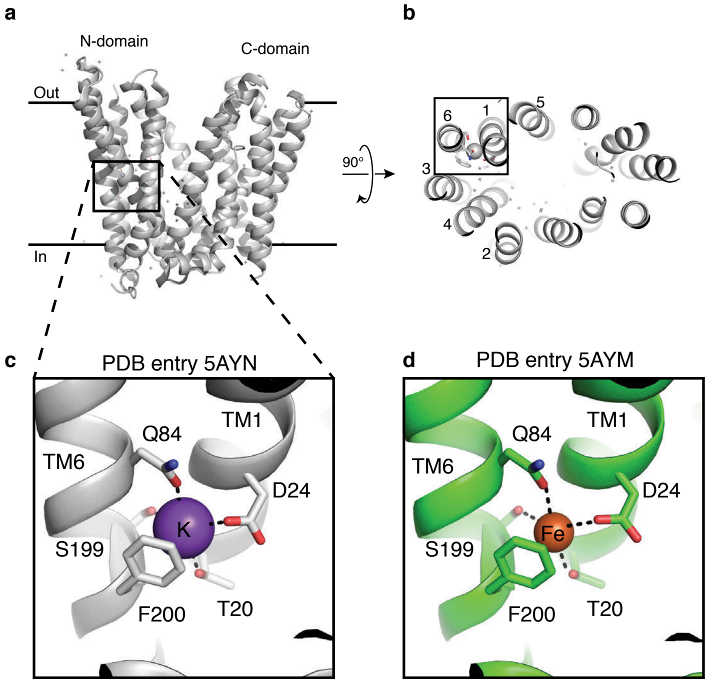


**Supplementary Figure 6. Cation binding site in previously determined BbFpn structures. a** Cartoon representation of previously determined outward facing conformation of BbFpn (PDB entry 5AYN). The location of the putative substrate binding site is indicated by rectangle box. **b** 5AYN structure viewed from the periplasmic side. Transmembrane (TM) helices 1-6 are numbered and the square box indicates the position of the cation-binding site (between TM1 and TM6), with the $K^+$ from 5AYN shown as a grey sphere. **c** Close up view of the cation binding site in 5AYN. The $K^+$ ion (from the crystallization condition) is coordinated by the residues shown in ball-and-stick. **d** Soaking with Fe (5 mM) gave a structure (PDB entry 5AYM) with anomalous difference density at the same site, suggesting Fe binding.

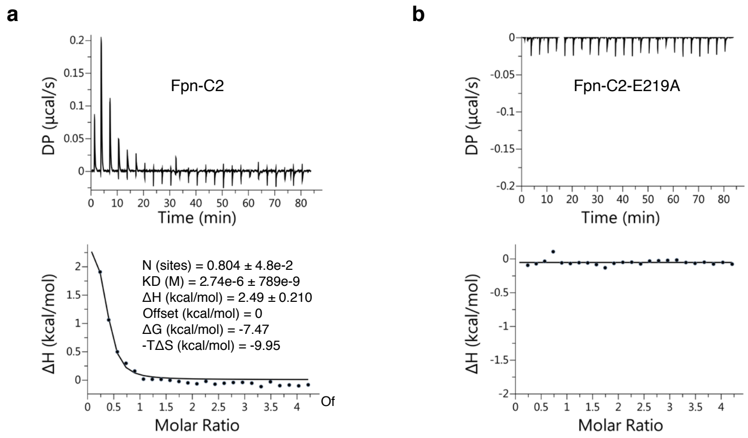


**Supplementary Figure 7. Isothermal titration calorimetry data. a** ITC isotherms of $Ca^{2+}$ to the thermostabilized mouse Fpn-C2 construct. **b** ITC measurements of $Ca^{2+}$ binding to Fpn-C2-E219A.

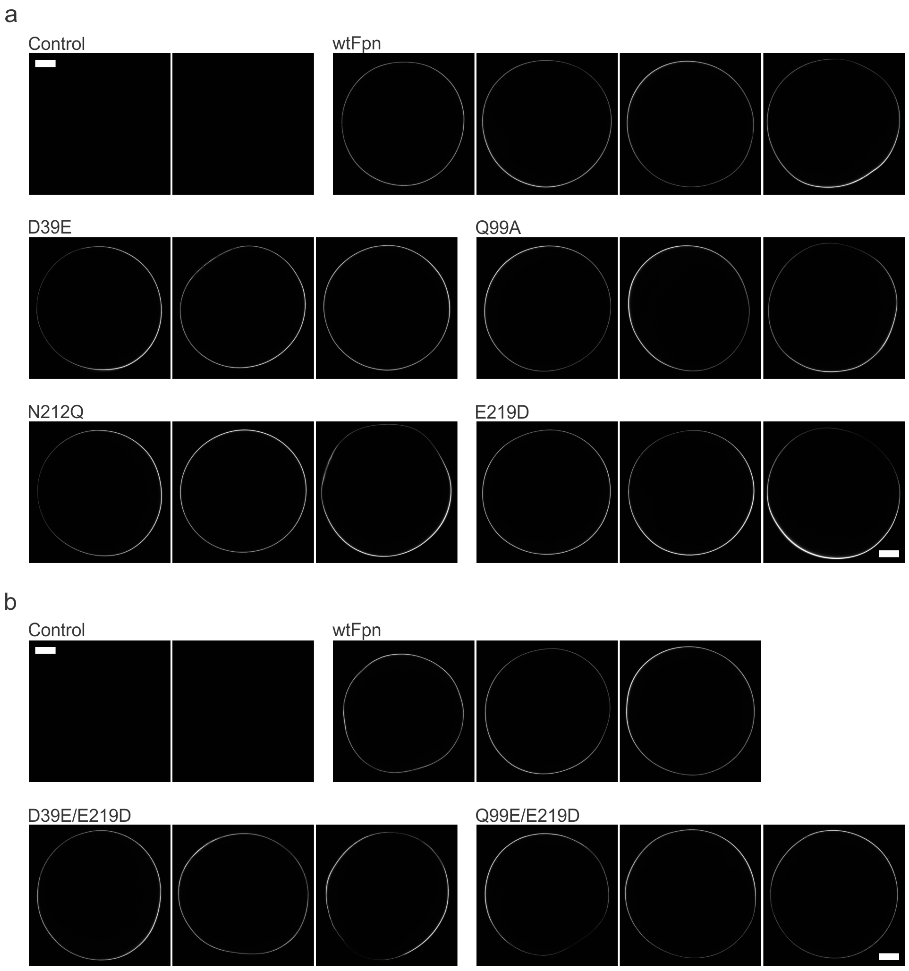


**Supplementary Figure 8. Mutagenesis of putative $Ca^{2+}$-coordinating residues in human Fpn.** Live-cell imaging of EGFP-tagged wildtype and mutant Fpn. Each image is of a separate oocyte mounted on the confocal LSM. All images were collected using identical settings. Scale bars, 0.2 mm.

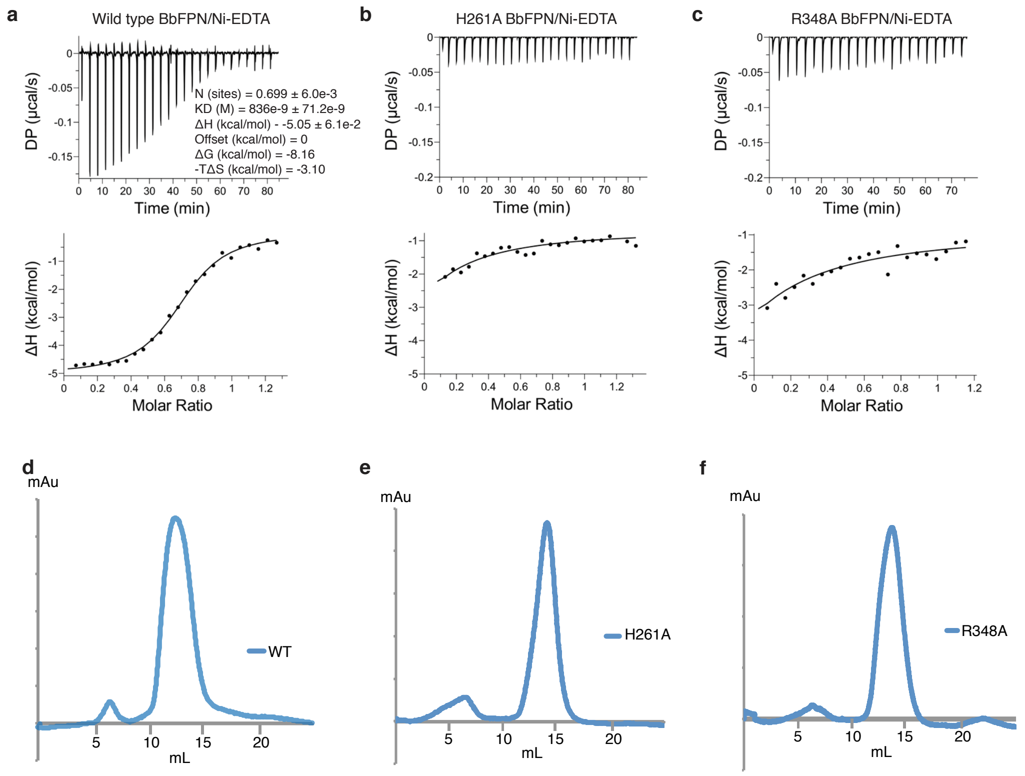


**Supplementary Figure 9. Isothermal titration calorimetry data. a-c** ITC isotherms of Ni-EDTA binding to wild-type BbFpn, H261A, and R348A, respectively. **d-f** Size exclusion chromatograms of wild type BbFpn and the mutants H261A and R348A. Samples were run on a Superdex 200 10/300GL (GE Lifesciences).

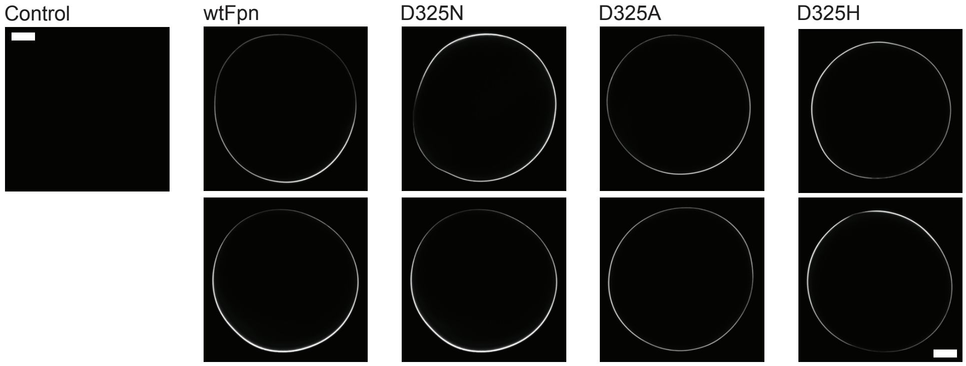


**Supplementary Figure 10. Live-cell imaging of oocytes expressing mutants of the putative Fe-binding site of human Fpn.** Each image is of a separate oocyte mounted on the confocal LSM. All images were collected using identical settings. Scale bars, 0.2 mm. a, Mutants in which we substituted D325 with N, A, or H, compared with wtFpn in the same oocyte preparation as used in Figure 6E.

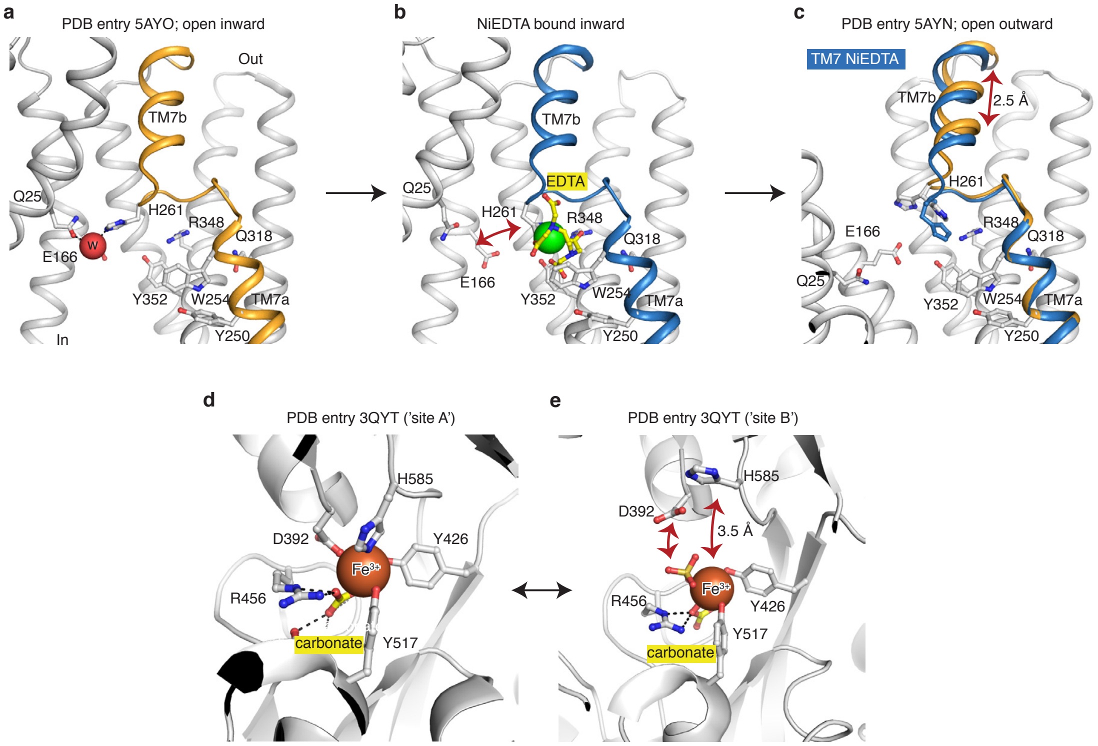


**Supplementary Figure 11. Proposed substrate binding and release mechanism by Fpn proteins. a** Open 'apo' inward conformation (PDB entry 5AYO) of BbFpn viewed parallel to the membrane normal. TM7 is shown in orange and discussed residues are shown in ball-and-stick representation. In this conformation, H261 is oriented away from the substrate-binding pocket, coordinating a water molecule (red sphere) together with Q25 and E166. In this state, the pocket is fully open and accessible for substrate binding. **b** Ni-EDTA bound structure, with TM7 shown in blue. Substrate binding leads to a conformational change in H261, which form a direct ligand to $Ni^{2+}$ (green sphere). **c** Open outward-facing structure (PDB entry 5AYN) with TM7 highlighted in orange. The C-terminal domain from the (Ni-EDTA bound) inward-facing conformation was superimposed and TM7 visualised in blue colour. The superimposition illustrates a shift in TM7b along the membrane normal. We propose that this shift disrupts the metal coordination by H261, which promotes release of both metal and metabolite. **d** Analogous substrate release can be seen in transferrin, in which carbonate and $Fe^{3+}$ binds synergistically. In transferrin, a conserved arginine residue (R456) coordinates the carbonate molecule, which in turn coordinate $Fe^{3+}$ together with surrounding residues. **e** In the substrate release mechanism, the $Fe^{3+}$ ligands D392 and H585 move away from the metal, promoting substrate release.

**Supplementary Table 1**. Primers used in study.

| Construct Name | Primer name | Primer sequence (5' to 3') |
|---|---|---|
| BbFPN | BbFPN-F | AAA ACT CGA GAT GAA AGT CCA GAG CTT |
| BbFPN | BbFPN-R | TTT TGG ATC CGG ATT CAA CGG GTT CAG CA |
| BbFpn-D24A | D24A-F | CCAGATCCGGGGCTCAGGCCTGGG |
| BbFpn-D24A | D24A-R | TCCCAGGCCTGAGCCCCGGATCTGG |
| BbFpn -Q84A | Q84A-F | TGGGGAGTCTGGCTGGCGTTCTTTGCCATTC |
| BbFpn -Q84A | Q84A-R | CAAGAATGGCAAAGAACGCCAGCCAGACTCCCC |
| BbFpn -Q84N | Q84N-F | AAGTGGGGAGTCTGGCTGAATTTCTTTGCCATTCTTGCC |
| BbFpn -Q84N | Q84N-R | GGCAAGAATGGCAAAGAAATTCAGCCAGACTCCCCACTT |
| BbFpn -Q84E | Q84E-F | TGGGGAGTCTGGCTGGAGTTCTTTGCCATTCTTG |
| BbFpn -Q84E | Q84E-R | GAATGGCAAAGAACTCCAGCCAGACTCCCC |
| BbFpn -N196A | N196A-F | GTTCCTGATTGGTCTTTGGGCTCTGGTTTCTTTTG |
| BbFpn -N196A | N196A-R | ATTCAGGAACAAAAGAAACCAGAGCCCAAAGACCAATC |
| BbFpn -E203A | E203A-F | AATCTGGTTTCTTTTGTTCCTGCATACTTTCTTTTGCGGAATGTG |
| BbFpn -E203A | E203A-R | CACATTCCGCAAAAGAAAGTATGCAGGAACAAAAGAAACCAG |
| hFpn-D39E | D39E-F | GCCACATCCGCTCTCCCCAAGTAGAGAGAG |
| hFpn-D39E | D39E-R | CTCTCTCTACTTGGGGAGAGCGGATGTGGC |
| hFpn-Q99E | Q99E-F | GACTGAAACATTCTCTACCACCAGCGAGGTCTG |
| hFpn-Q99E | Q99E-R | CAGACCTCGCTGGTGGTAGAGAATGTTTCAGTC |
| hFpn-Q99A | Q99A-F | AGGATGACTGAAACATTCGCTACCACCAGCGAGGTCTG |
| hFpn-Q99A | Q99A-R | CAGACCTCGCTGGTGGTAGCGAATGTTTCAGTCATCCT |
| hFpn-N212Q | N212Q-F | GGCTTTATTTCGGGATGGCAGTTGGTATCCATGTGCGTG |
| hFpn-N212Q | N212Q-R | CACGCACATGGATACCAACTGCCATCCCGAAATAAAGCC |
| hFpn-E219A | E219A-F | GTGTCCATGTGTGTGGCGTACTTCTTGCTCTGG |
| hFpn-E219A | E219A-R | CCAGAGCAAGAAGTACGCCACACACATGGACAC |
| hFpn-E219D | E219D-F | GTATCCATGTGCGTGGATTACGTTCTGCTCTGGAA |
| hFpn-E219D | E219D-R | TTCCAGAGCAGAACGTAATCCACGCACATGGATAC |
| hFpn-D325A | D325A-F | CTGTCCTGGGCTTTGCCTGCATCACCACAGG |
| hFpn-D325A | D325A-R | CCTGTGGTGATGCAGGCAAAGCCCAGGACAG |
| hFpn-D325H | D325H-F | ATATGACTGTCCTGGGCTTTCACTGCATCACCAC |
| hFpn-D325H | D325H-R | GTGGTGATGCAGTGAAAGCCCAGGACAGTCATAT |
| hFpn-D325N | D325N-F | ATATGACTGTCCTGGGCTTTAACTGCATCACCAC |
